# Trichoblastic carcinosarcoma with panfollicular differentiation (panfollicular carcinosarcoma) and *CTNNB1* (beta‐catenin) mutation

**DOI:** 10.1111/cup.13794

**Published:** 2020-07-20

**Authors:** Jenny Giang, Asok Biswas, Antien L. Mooyaart, Floris H. Groenendijk, Petra Dikrama, Jeffrey Damman

**Affiliations:** ^1^ Department of Pathology Erasmus University Medical Center Rotterdam The Netherlands; ^2^ Department of Pathology, Western General Hospital University of Edinburgh Edinburgh UK; ^3^ Department of Dermatology Erasmus University Medical Center Rotterdam The Netherlands

## Abstract

We present a case of trichoblastic carcinosarcoma with panfollicular differentiation. An 80‐year‐old man presented with a lesion on the left ear, which had been present for several months. Histopathology revealed a well‐demarcated neoplasm in the dermis composed of intimately intermingled malignant epithelial and mesenchymal cells. The epithelial component showed multilineage follicular differentiation toward all of the elements of a normal hair follicle. Molecular analysis revealed identical molecular aberrations in both epithelial and mesenchymal components including *CTNNB1* and *SUFU* mutations. To the best of our knowledge, this is the first report of panfollicular carcinosarcoma and of the presence of a *CTNNB1* mutation in trichoblastic carcinosarcoma.

## 1 INTRODUCTION

Trichoblastic carcinosarcoma is an exceedingly rare biphasic cutaneous malignant neoplasm featuring a mixture of epithelial elements, resembling follicular germinative cells, along with mesenchymal component. Only eight cases of trichoblastic carcinosarcoma have been reported in the literature.^1^ A few cases of trichoblastic carcinosarcoma showed differentiation toward various elements of the hair follicle, for example, clear‐cell change in epithelial aggregates and trichohyalin keratinization.^2^, ^3^ In contrast to trichoblastic carcinoma, multiple paths of hair follicle differentiation or panfollicular differentiation have not been reported in trichoblastic carcinosarcoma.^4^ In this report, we present a unique case of trichoblastic carcinosarcoma with panfollicular differentiation (or panfollicular carcinosarcoma). In addition, we investigate the molecular changes underlying this neoplasm.

## 2 CASE REPORT

**FIGURE 1:**
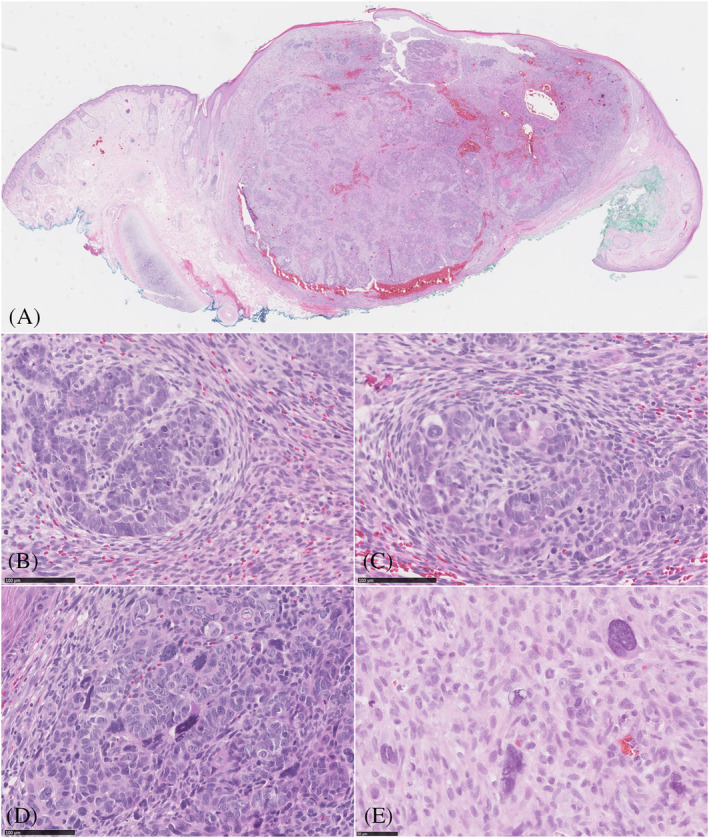
Scanning magnification demonstrates a biphasic cutaneous tumor (A, H&E magnification 61×), the tumor consisted of an epithelial follicular germinative cell component intimately intermingled with the mesenchymal component (B,C, H&E magnification 200×), epithelial component composed of atypical cells with polymorphic and pleomorphic nuclei (D, H&E magnification 200×), also note the multinucleated pleomorphic cells with prominent nucleoli dispersed between the spindle cells (E; H&E magnification 200×)

**FIGURE 2:**
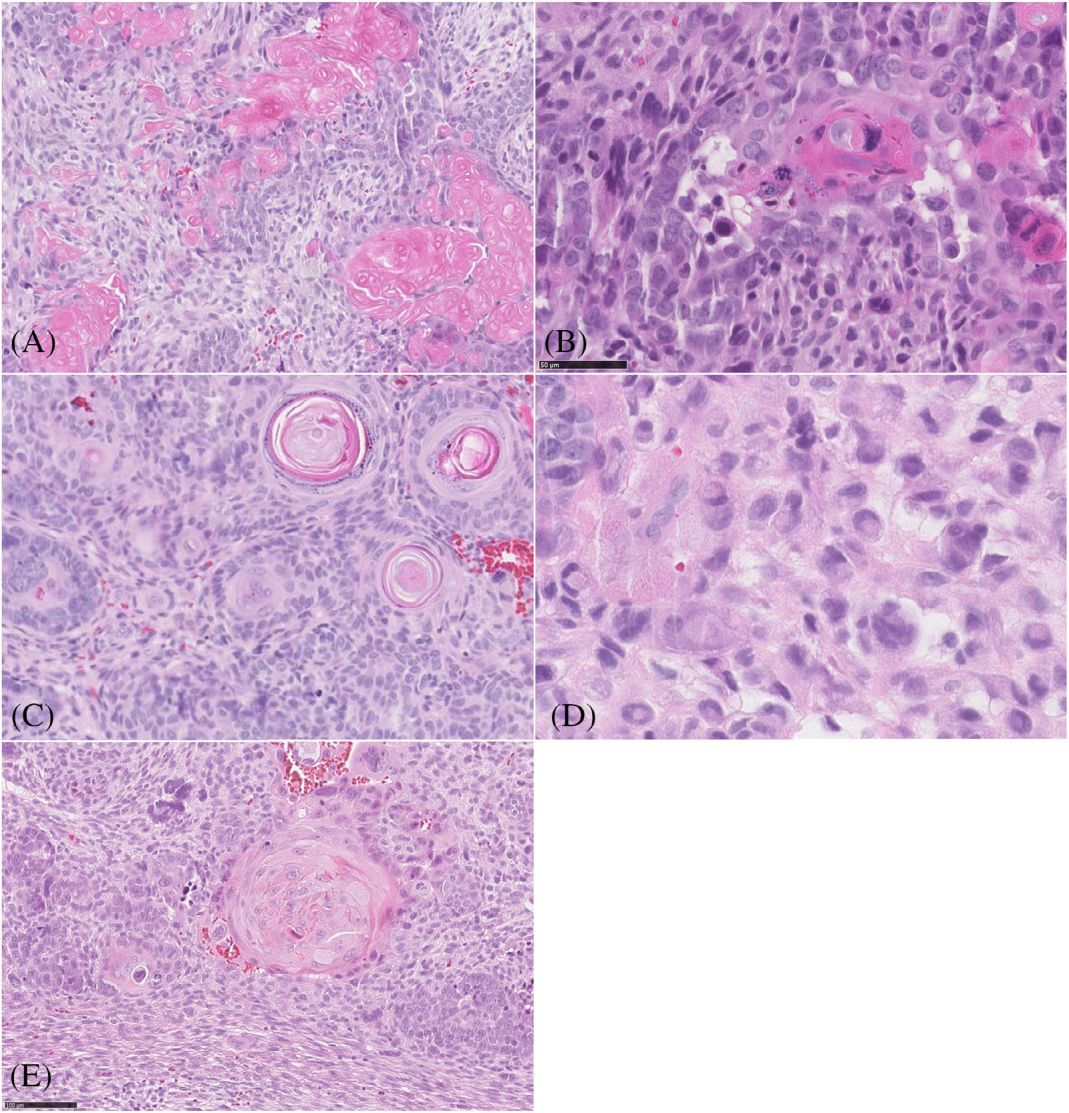
Figure 2 A‐D, Histopathological features of the various differentiation: inner root sheath differentiation of the bulb and stem with ghost/matrical cells (A, H&E magnification 100×), and bright red trichohyalin granules; (B, H&E magnification 200×). Infundibular differentiation with infundibulocystic and squamous structures surrounded by mesenchymal cells (C, H&E magnification 120×). Cells with signet ring cell appearance (myoepithelial differentiation) (D, H&E magnification 200×). Eosinophilic cells with central apoptosis (trichilemmal differentiation of outer root sheath) (E, H&E magnification 100×)

An 80‐year‐old man with no relevant past medical history presented with a lesion on the left ear, which had been present for a few months. Histopathology revealed a well‐demarcated neoplasm in the dermis without subcutaneous involvement or connection to the epidermis (Figure 1). The lesion was composed of intimately intermingled malignant epithelial and mesenchymal cells. The vast majority of the tumor consisted of an epithelial follicular germinative cell component arranged in nodular, retiform, and petaloid patterns composed of atypical basaloid cells. The cells were relatively small with high nuclear‐cytoplasmic ratio, and had oval vesicular pleomorphic nuclei, sometimes with a prominent nucleolus. Mitotic figures were conspicuous. Throughout the lesion, the epithelial component showed multilineage follicular differentiation toward all of the elements of a normal hair follicle (Figure 2). Upper and lower hair segment differentiation was represented by the formation of keratocysts and infundibulocystic structures (infundibular/upper isthmic differentiation), ghost/matrical cells and bright red trichohyalin granules (inner root sheath differentiation of the bulb and stem), and focal presence of eosinophilic cells with central apoptosis (trichilemmal differentiation of the outer root sheath). In addition, intracellular hyaline globules, foci of calcification, and epithelial structures resembling hair papillae were seen and signet ring cell differentiation was present (myoepithelial differentiation). The mesenchymal component was intimately intermingled with the epithelial component and showed an organoid growth pattern with papillary mesenchymal bodies. On the one hand, the spindle cells showed indistinct cytoplasmic borders, round‐oval to elongated nuclei and small nucleoli. However, dispersed throughout the spindle cells, multinucleated pleomorphic cells with prominent nucleoli were also present.

**FIGURE 3:**
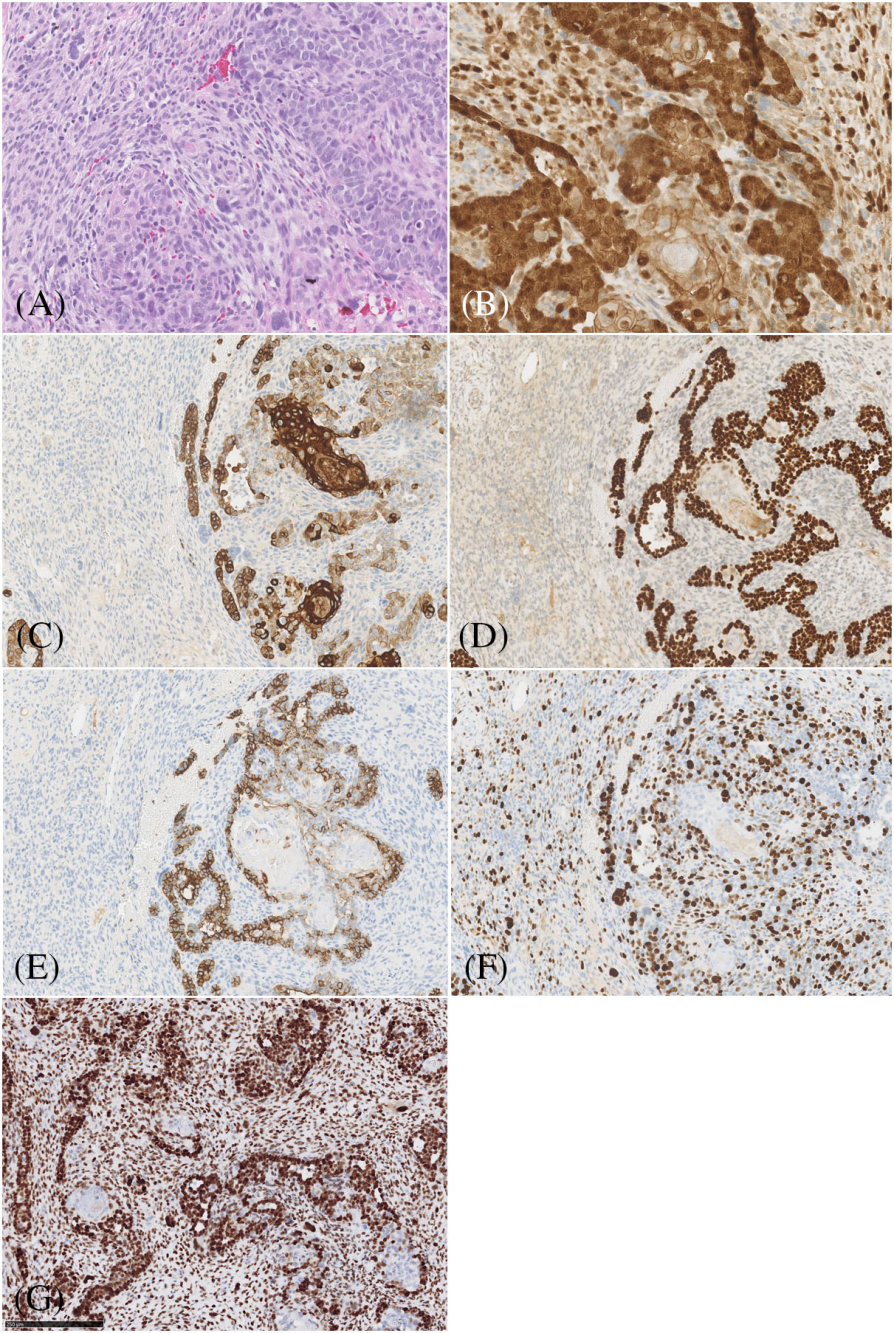
Figure 3 A‐G, Area with epithelial and mesenchymal component (A, H&E magnification 100×), strong nuclear beta‐catenin expression was noted in both epithelial and mesenchymal cells (B, magnification 100×), cytokeratin AE1/AE3 showed strong expression in epithelial cells and negative staining in mesenchymal cells. (C, magnification 50×), p63 were strongly expressed in malignant epithelial cells, while negative in mesenchymal cells (D, magnification 50×), BerEP4 showed focally weak expression in the malignant epithelial cells (E, magnification 50×), Ki‐67 proliferation was high in both components (F, magnification 50×), aberrant expression of p53 was demonstrated in both components (G, magnification 50×)

Immunohistochemically, the epithelial component was positive for cytokeratin (CKAE1/AE3), BerEp4 (partially), and p63. Aberrant expression of p53 was demonstrated in both components. Interestingly, both the epithelial and mesenchymal components revealed focal strong nuclear beta‐catenin staining, mainly in inner root cells, matrical cells, and spindle cells. Ki‐67 proliferation index was high (30%) in both components and accentuated the nuclear polymorphism in both components (Figure 3).

Molecular analysis using targeted next‐generation sequencing (NGS) of cancer‐associated genes and single‐nucleotide polymorphism (SNP) analysis for copy number alterations was performed using two different gene panels (Supporting Information). Separate areas of epithelial and mesenchymal components were microdissected under a stereomicroscope (ZEISS SteREO Discovery.V8). There was no overlap between the two components. Analysis revealed identical mutations in both elements, that is, *TERT* promoter and *TP53* mutation, an activating mutation in *CTNNB1* (encoding beta‐catenin) and truncating mutations in both *CDKN2A* (encoding p16/p14arf) and *SUFU* (a negative regulator of Hedgehog signaling) (Table 1). Mutations in *CTNNB1* and *SUFU* were present in both components with a lower variant allele frequency than expected based on the tumor cell percentage, indicative for subclonal presence of both mutations. Additionally, identical copy number alterations including imbalance of chromosomes 3p, 4q, and 19 and loss of 5q, 9p, 10, 13q, and 17 were identified in both components. Mutations in *PTCH1* and *SMO*, the key components of the Hedgehog pathway, commonly involved in basal cell carcinoma carcinogenesis, were not found in this tumor.

**TABLE 1:** Mutations and corresponding variant allele frequencies (VAF) in both tumor components

	Epithelial component	Mesenchymal component
	Estimated tumor cell percentage 80%	Estimated tumor cell percentage 70%
TP53 (NM_000546) c.613T>A; p.Y205N	87%	64%
CDKN2A (NM_000077) c.172C>T; p.R58*	91%	55%
TERT promoter C228T (NC_000005.10:g.1295228G>A (Chr5, Hg19)	58%	54%
CTNNB1 (NM_001098209) c.134_135delinsTG; p.S45L	31%	15%
SUFU (NM_016169) c.847delinsCA; p.E283Qfs*3	34%	21%

## 3 DISCUSSION

Trichoblastic carcinosarcoma is an exceedingly rare tumor. To date, only eight cases have been reported in the literature.^1^ Our report is the first case to describe panfollicular differentiation within trichoblastic carcinosarcoma. Due to the rarity and underreporting of trichoblastic carcinosarcoma, knowledge of the exact etiology is limited. Several hypotheses have been proposed to explain biphasic tumors: two distinct tumors connecting (collision tumor), divergent differentiation originating from a single progenitor cell toward epithelial and mesenchymal cells (monoclonal), and multiclonal origin.^5^ Our data strongly support the monoclonal origin of both components, in line with a previous study that showed similar genetic and chromosomal aberrations in epithelial and mesenchymal components in cutaneous carcinosarcoma.^6^

Interestingly, using immunohistochemistry, we found nuclear beta‐catenin staining in parts of both the epithelial and mesenchymal components. Indeed, additional molecular analysis confirmed a *CTNNB1* mutation (exon 3: c.134_135delinsTG; p.S45L). This specific mutation in Serine 45 has not been reported earlier, although Serine 45 is a mutational hotspot in other tumor types and different mutations in this residue have been shown to be oncogenic/activating. Mutations in exon 3 are associated with translocation of the beta‐catenin protein from the membrane to the nucleus and activation of Wnt/beta‐catenin signaling. Aberrant expression of beta‐catenin has been reported in tumors with matrical differentiation, such as pilomatricoma, pilomatrix carcinoma, and basal cell carcinoma with matrical differentiation. Furthermore, Kazakov et al described one case of a trichoblastoma harboring a *CTNNB1* mutation. To our knowledge, this is the first report of a trichoblastic carcinosarcoma harboring a *CTNNB1* mutation.^7^ Wnt/beta‐catenin signaling seems to play a role in multiple processes. Firstly, it has been shown to be involved in hair follicle development and differentiation.^8^, ^9^ Secondly, beta‐catenin may regulate stem cell pluripotency since *Wnt* activation leads to binding of beta‐catenin to transcription factors resulting in pluripotent gene regulation.^10^ Gat et al demonstrated that stabilized beta‐catenin in mice undergoes a process leading to de novo hair follicle morphogenesis, and they suggest that aberrant beta‐catenin activation results in follicular tumors.^11^ In the case of trichoblastic carcinosarcoma, it is likely that dysregulation of *Wnt* signaling leads to dysregulated follicular differentiation.

The Hedgehog signaling pathway is essential for embryonic development and plays a crucial role in tumorigenesis. The *SUFU* protein acts as a negative regulator of the Hedgehog signaling pathway by binding to and inhibiting *Gli1* protein. Inactivating mutations in *SUFU* can result in upregulation of *Gli* transcription factors leading to aberrant activation of the Hedgehog pathway. Furthermore, Hedgehog signaling regulates the growth and morphogenesis of hair follicle epithelium during the anagen portion of the hair cycle.^12^ Our case showed *SUFU* mutation in both components of the tumor. Mutations in *SUFU* gene have been associated with Gorlin syndrome, multiple hereditary infundibulocystic basal cell carcinoma syndrome, familial meningioma, and medulloblastoma.^13^ However, it should be noted that, unlike in our case, the abovementioned were germline *SUFU* mutations.

According to the literature, trichoblastic carcinosarcoma are more frequently seen in older individuals, with male predominance, and are mainly located in the head and neck region, as in our case. Trichoblastic carcinosarcoma has not been reported in young adults or children.^1^, ^2^

No guidelines regarding treatment and follow‐up of trichoblastic carcinosarcoma have been established because of the rarity of these tumors. In our patient, re‐excision with Mohs surgery was performed since the tumor was found close to the resection margin in the first excision. Although the follow‐up period is limited, there has been no local recurrence over a period of 6 months. There were no lymph node metastases.

In conclusion, we describe the first case of trichoblastic carcinosarcoma with panfollicular differentiation and identify identical molecular changes, including beta‐catenin and *SUFU* mutations, in the epithelial and mesenchymal components, suggesting that trichoblastic carcinosarcoma might originate from a single progenitor cell (monoclonal hypothesis). This finding may shed light on the underlying biology of this tumor type. However, further investigation including further molecular studies is needed for a better understanding of trichoblastic carcinosarcoma.

## Supporting information


**Appendix** **S1**: Supporting information
