# Supplementary material for: Trichoblastic carcinosarcoma with panfollicular differentiation (panfollicular carcinosarcoma) and CTNNB1 (beta‐catenin) mutation
Source: J Cutan Pathol. 2020 Jul 20;48(2):309–13. doi: 10.1111/cup.13794 (PMC7891381; doi:10.1111/cup.13794)
Supplement: Supplementary file 1 — Appendix S1: Supporting information [file CUP-48-309-s001.docx]

**Supplementary information. Content NGS panels**

**Content NGS panel 1**

Coding sequence: CDKN2A (coverage based on design 98%), PTEN (94%) and TP53 (100%)
Mutational hotspots: AKT1 (exon 3), ALK (20, 22-25), APC (14), ARAF (7), BRAF (11, 15), CTNNB1 (3, 7, 8), EGFR (18-21), HER2 (19-21), EZH2 (16), FBWX7 (9, 10), FGFR1 (4, 7, 12), FGFR2 (7, 9, 12), FGFR3 (7, 9), FOXL2 (1), GNA11 (4, 5), GNAQ (4, 5), GNAS (8, 9), HRAS (2-4), IDH1 (4), IDH2 (4), KIT (8, 9, 11, 13, 14, 17), KRAS (2-4), MAP2K1 (2, 3), MET (2, 14, 19), MYD88 (5), NOTCH1 (26, 27), NRAS (2-4), PDGFRA (12, 14, 18), PIK3CA (10, 21), POLD1 (12), POLE (9, 13), RAF1 (7), RET (11, 16), RNF43 (3, 4, 9), ROS1 (38, 41), SMAD4 (3, 9, 12), STK11 (4, 5, 8)
Non-coding sequence: TERT promoter

**Content NGS panel 2**

Coding sequence: ATRX (coverage based on design 97%), CDKN2A (94%), CDKN2B (91%), CIC (81%), DAXX (93%), DDX3X (95%), FUBP1 (97%), NF1 (99%), NF2 (100%), PTCH1 (96%), PTCH2 (92%), PTEN (94%), SETD2 (96%), SMO (87%), SUFU (97%), TRAF7 (72%) and TP53 (98%).

Mutational hotspots: ACVR1 (exon 6-9 ), AKT (3), ALK (20, 22-25), BRAF (11, 15), CTNNB1 (3), EGFR (exon 3, 7, 15), FGFR1 (12, 14, 15), H3F3A (2), H3F3B (2), HIST1H3B (1), HIST1H3C (1), IDH1 (4), IDH2 (4), KLF4 (4) and PIK3CA (10, 21).

Non-coding sequence: TERT promoter
